# The proportion of alveolar type 1 cells decreases in murine hypoplastic congenital diaphragmatic hernia lungs

**DOI:** 10.1371/journal.pone.0214793

**Published:** 2019-04-17

**Authors:** Tram Mai Nguyen, Julio Jimenez, Linda Elowsson Rendin, Catharina Müller, Gunilla Westergren-Thorsson, Jan Deprest, Jaan Toelen

**Affiliations:** 1 Department of Development and Regeneration, Division Organ Systems, KU Leuven, Leuven, Belgium; 2 Lung Biology, Department of Experimental Medical Science, Lund University, Lund, Sweden; 3 Department of Obstetrics and Gynaecology, Division Woman and Child, University Hospitals Leuven, Leuven, Belgium; 4 Institute for Women’s Health, University College London, London, United Kingdom; 5 Department of Paediatrics, Division Woman and Child, University Hospitals Leuven, Leuven, Belgium; Children's Hospital of Los Angeles, UNITED STATES

## Abstract

**Background:**

Pulmonary hypoplasia, characterized by incomplete alveolar development, remains a major cause of mortality and morbidity in congenital diaphragmatic hernia. Recently demonstrated to differentiate from a common bipotent progenitor during development, the two cell types that line the alveoli type 1 and type 2 alveolar cells have shown to alter their relative ratio in congenital diaphragmatic hernia lungs.

**Objective:**

We used the nitrofen/bisdiamine mouse model to induce congenital diaphragmatic hernia and accurately assess the status of alveolar epithelial cell differentiation in relation to the common bipotent progenitors.

**Study design:**

Pregnant Swiss mice were gavage-fed with nitrofen/bisdiamine or vehicle at embryonic day 8.5. The administered dose was optimized by assessing the survival, congenital diaphragmatic hernia and facial abnormality rates of the exposed mouse pups. NanoCT was performed on embryonic day 11.5 and 16.5 to assess the embryonic and early canalicular stages of lung development. At embryonic day 17.5 corresponding to late canalicular stage, congenital diaphragmatic hernia lungs were characterized by measuring the lung weight/body weight ratio, morphometry, epithelial cell marker gene expression levels and alveolar cell type quantification.

**Results:**

Nitrofen/bisdiamine associated congenital diaphragmatic hernia lungs showed delayed development, hypoplasia with morphologic immaturity and thickened alveolar walls. Expression levels of distal epithelial progenitor marker Id2 increased, alveolar type 1 cell markers Pdpn and Hopx decreased, while type 2 cell markers pro-SPC and Muc1 remained constant during the canalicular stage. The number of Pdpn^+^ type 1 alveolar cells also decreased in congenital diaphragmatic hernia lungs.

**Conclusion:**

The mouse nitrofen/bisdiamine model is a potential model allowing the study of congenital diaphragmatic hernia lung development from early stages using a wide array of methods. Based on this model, the alveolar epithelium showed a decrease in the number of alveolar type 1 cell in congenital diaphragmatic hernia lungs while type 2 cell population remains unchanged.

## Introduction

Congenital diaphragmatic hernia (CDH) is a structural anomaly characterized by variable degrees of defects in the diaphragm. During pregnancy, abdominal contents enter the chest via the defect, leaving insufficient space for normal lung development. The resulting pulmonary hypoplasia remains one of the critical factors determining the outcomes in CDH newborns [1–3]. CDH lungs show parenchymal underdevelopment with delay of alveolar growth, fewer bronchial branches and alveoli [4,5]. The pathogenesis of lung hypoplasia related to CDH is still not fully comprehended.

Further insights into the pathophysiology of CDH necessitate specific animal models. These have been developed using surgical, teratogenic, and genetic techniques. The earliest models relied on surgically induced diaphragmatic defects in fetuses that had, until that moment, normal lungs and diaphragm [6–8]. These, therefore, are less informative about the etiology and mechanisms responsible for the early-onset disturbed lung development. On the other hand, the genetic models have added extra information about CDH pathophysiology [7,9,10]. A number of gene mutations which induce diaphragmatic defects have been identified in mice. However, disruption of these genes explains only a minority of CDH cases, leaving a large number of genetic abnormalities yet to be identified. Another widely used CDH model is the teratogenic nitrofen (2,4-dichloro-4′-nitrodiphenyl ether) model [7,11]. Nitrofen is a herbicide that induces congenital anomalies in rodent fetuses. Depending on the dosage, timing, and species, the anomalies can include diaphragmatic defects, pulmonary hypoplasia, immaturity, and vascular anomalies [11–14]. Other chemicals have also been identified as teratogens causing CDH including bisdiamine or N,N′-octamethylenebis (dichloroacetamide). Bisdiamine is a spermatogenesis inhibitor which can cause CDH in rats [14,15]. A combination of nitrofen and bisdiamine (N/B) has been shown to induce CDH in mice [16]. This model enables researchers to comprehensively study CDH lung development at molecular, tissue and system levels.

The main function of the mammalian lung is gas exchange between the blood and the external environment. The two major alveolar epithelial cell (AEC) types that constitute the gas exchange compartment are alveolar type 1 (AT1) and type 2 (AT2) cells [17–22]. In CDH-associated lung hypoplasia, the ratio of AECs is known to be altered, which was thought to be due to the differentiation of AT2 into AT1 cells [23,24]. Although the histological abnormalities in CDH lungs have been well described, less is known about the underlying spatiotemporal differentiation patterns and their molecular mechanisms. Recent studies reveal a population of bipotent progenitors (BP) expressing markers of both AT1 and AT2 cells. These BPs differentiate into mature AT1 or AT2 cells by upregulating markers of the corresponding cell fate, and downregulating markers of the alternative cell fate during lung development [25,26]. As other CDH models in previous studies did not address their presence, visualizing BPs together with the differentiated alveolar cells could give a more detailed insight into the alveolar cell numbers and fates in CDH. To allow spontaneous monitoring of multiple cell types, a murine model is ideal as the number of cellular markers of interest have been identified in mice.

We used a mouse teratogenic model to accurately study the differentiation pattern of AECs in CDH lungs with regards to BPs. The dissection of the pathophysiological mechanisms that govern cell fate in CDH may enable the development of novel strategies in the treatment of CDH and the associated pulmonary hypoplasia.

## Materials and methods

### The teratogenic CDH mouse model

Time-mated pregnant CD1 mice were provided by the Animalium at KU Leuven. Females caged with males were checked the morning after mating for the presence of a vaginal plug as an indication of mating. Noon of the day on which a vaginal plug was detected was considered E0.5. All procedures involving animals and the study protocol were approved by the Ethics Committee for Animal Experimentation of the Faculty of Medicine, KU Leuven (project number: P033/2016).

To induce CDH, 15 mg of 2,4-Dichloro-1-(4-nitrophenoxyl) benzene (nitrofen) (RDP00053EB, Thermo Fisher Scientific) and 0–10 mg of N,N’- Octamethylenebis (2,2- dichloroacetamide) (bisdiamine) (sc-295819A, Santa Cruz Biotechnology) dissolved in olive oil (O1514, Sigma) was gavage fed as a single dose under light anesthesia (2 min of Isoflurane 2.5%) at E8.5 [16]. In randomly allocated control animals, the same volume of vehicle (olive oil or OO) was given without N/B. The mice were euthanized at E11.5, E16.5, or E17.5. All fetuses were delivered by cesarean section. Embryos were harvested by cesarean section and anesthetized by hypothermia on ice. Then the lungs were carefully dissected, weighed and either fixed in formaldehyde solution 4% before embedding in paraffin or snap frozen in liquid nitrogen in accordance with the planned subsequent experiments.

**Fig 1:**
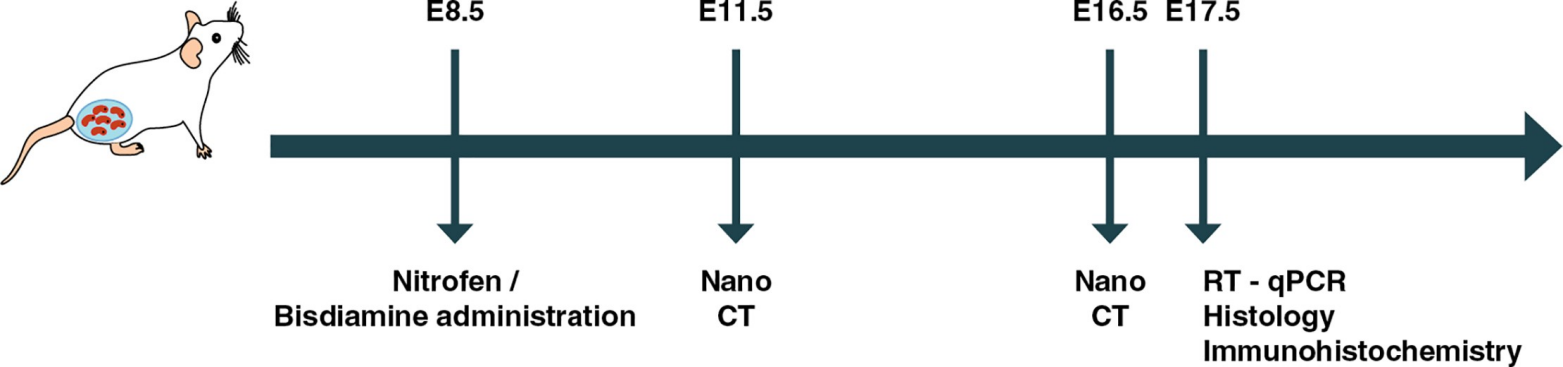
Schematic depicting time points for congenital diaphragmatic hernia induction by nitrofen/bisdiamine (N/B) and lung harvesting for specific assessment methods. Pregnant CD1 mice were gavage-fed with N/B on E8.5. For nanoCT assessment, whole pups were harvested on either E11.5 or E16.5. For RT-qPCR analysis, histology or immunohistochemistry analysis, fetal mouse lungs were harvested on E17.5.

Embryonic lungs were allocated into two groups for analysis: CDH group (from pups confirmed as having N/B-induced CDH with an observed defect in the diaphragm) and control group (from pups of which the mothers were fed with vehicle). The experimental timeline is shown in Fig 1.

### Immunohistochemistry

Lungs from E17.5 embryos were fixed in formaldehyde solution 4% (4078–9001, Klinipath) at room temperature for 2 hours, washed in phosphate buffered saline (PBS) for 1 hour and later embedded in paraffin blocks. 4 μm-thick whole lung sections were processed following the standard deparaffinization and immunostaining protocol. Primary antibodies used were as follows: rabbit anti-proSPC (1:900, Abcam Cat# ab90716, RRID:AB_10674024), and hamster anti-Pdpn (1:1000, Abcam Cat# ab11936, RRID:AB_298718). The following secondary antibodies were used at 1:500: Goat anti-hamster; Alexa Fluor 488 (ab180063, Abcam); Goat anti-rabbit; Alexa Fluor 555 (A-21430, Thermo Fisher Scientific). Nuclei were either stained with Hoechst dye (B2261, Sigma) or DAPI from the Vectashield mounting medium (H1200, VectorLab Inc). Images were captured on a Zeiss LSM 880 (Oberkochen, Germany).

### Immunostaining quantification

Slides from either controls or mutants were immunofluorescently stained for pro-SPC and Pdpn. Three images were taken from similar areas of each slide so that the total number of counted cells exceeded 1000. For pro-SPC and Pdpn quantification, the number positive cells and DAPI+ cells in the entire field were semi-automatedly counted to determine the percentage of positive cells using QuPath software by utilizing the fast cell counting and positive cell detection functions [27]. Then the percentage of positive cells over the total of counted cells were evaluated for each sample.

### Quantitative RT-PCR

Total RNA was extracted from snap-frozen lungs using the Tripure isolation reagent (11667165001, Roche Diagnostics) according to the manufacturer’s protocol. Quantitative real-time polymerase chain reaction (RT-qPCR) was performed using Platinum SYBR Green qPCR SuperMix-UDG (11744500, R223-01, Thermo Fisher). Real-time qPCR was performed using a StepOnePlus Real-Time PCR thermal cycling block system (Applied Biosystems, Life Technologies). The mRNA levels of target genes were normalized to the *Hprt* mRNA level using the 2^−ΔΔCt^ method [28]. The error bars display the calculated maximum (RQMax) and minimum (RQMin) of the range of fold change which were calculated by incorporating the standard deviation of the ΔΔCt value into the fold difference calculation. Primers used for RT-qPCR are listed in S1 Table.

### Morphometry

Lungs were embedded in paraffin, and 4μm midsagittal sections were stained with hematoxylin and eosin. Sections were scanned with a slide scanner (AxioScan). Afterwards, a purpose-designed Fiji plug-in randomly selected 250x250μm fields. For each lung, the mean linear intercept of parenchymal airspace (Lma) and mean wall transactional length (Lmw) were calculated using a stereological tool software STEPanizer based on a previously described method based on overlapping a 36-point grid on 15 fields per lung [8,29–32]. Lma reflects the index of the size of the airspaces the air spaces and Lmw is an index of the thickness of alveolar septa.

### Nano-CT

N/B and OO administered pregnant CD1 mice were euthanized at E11.5 or E16.5 days by cervical dislocation. The embryos were dissected, washed with PBS and fixed in 4% paraformaldehyde overnight. Each sample was submerged in a 0.1 N iodine solution for 24 hours on a rotator at room temperature. Iodine solution was changed every 8 hours [33]. The nanoCT system utilized was a Phoenix NanoTom M (GE Measurement and Control Solutions, Germany) equipped with a Diamond-Tungsten target. E11.5 embryos were scanned at a voltage of 90 kV and a current of 180 μA. E16.5 embryos were scanned at a voltage of 50 kV and a current of 142 μA. The exposure time was 500 ms. Reconstruction was performed using Phoenix datos|x CT software. Representative images and videos of 3D volume renderings were created using CTvox software (Bruker, Belgium) (S1 File). Pseudocoloring of the lungs in the images was done using the free software GNU Image Manipulation program (GIMP 2.10).

### Statistical analysis

Statistical analysis of significance was calculated based on unpaired 2-tailed t-tests using SPSS 20.0 (IBM Corp., Armonk, NY) or GraphPad Prism 7.0 software (GraphPad Software, Inc., San Diego, CA). All data except RT-qPCR are presented as mean ± SEM (as indicated in the figure legends). *p<0.05, **p<0.01, ***p<0.001, ****p<0.0001 were considered statistically significant.

## Results

### Nitrofen/bisdiamine-induced CDH results in delayed lung development before the end of diaphragm formation

We tested different concentrations of administered N/B, including the combination that has earlier been shown to induce CDH in the mouse model [16]. These concentrations resulted in diaphragmatic defects, yet also consistently in the presence of facial abnormalities (S2 Fig). The latter has not been described previously in details. When given without bisdiamine, 15mg nitrofen resulted in 0% of CDH. Hence, we kept the nitrofen constant at 15mg while changing the bisdiamine concentration to investigate its dosage effects on survival, CDH and facial abnormality rates. Increased concentration of bisdiamine did not affect the survival rate. However, too low a concentration of bisdiamine (0 or 1mg) resulted in a low CDH rate (<0.2), while high doses (dose to be specified) were associated with a high rate of facial abnormalities (S2 Fig). Hence, we opted for a N/B combination of 15mg/3mg, which had the lowest rate of facial abnormalities while the CDH rate was still above 50%. In the N/B cohort, we only included the pups with an obvious diaphragmatic defect at necropsy. Additional abnormalities related to this dose were detected from the nanoCT scans by a trained clinician who is familiarized with interpreting CT scans (S2 Table).

**Fig 2:**
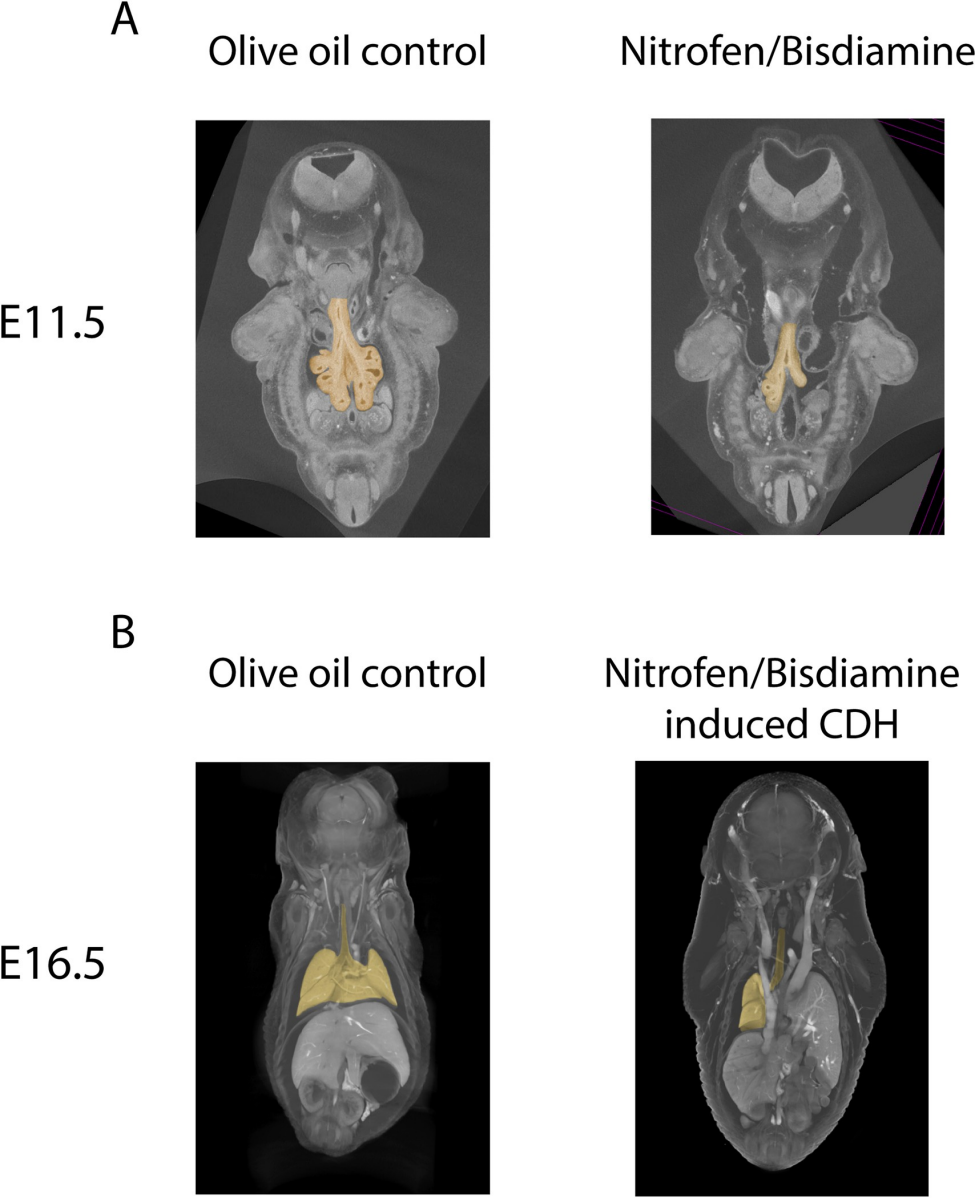
Nitrofen/bisdiamine caused lung development delay since embryonic stage. (A) Nano-CT sections of E11.5 (embryonic) and (B) E16.5 (early canalicular) mouse embryos allow visualizing lungs of nitrofen/bisdiamine (N/B) pups with regards to other organs. Lungs are pseudocolored in yellow. (A) Lungs showed delayed development at early embryonic stage when diaphragm has not finished its formation in a N/B pup compared to olive oil (OO) control. (B) At E16.5, N/B can induce CDH pup with diaphragmatic hernia through which the liver herniates into the thoracic cavity.

Nano-CT analysis was performed at either embryonic (E11.5) or early canalicular stage (E16.5). At E11.5, when the diaphragm has not yet finished its formation, N/B-exposed pups showed a delay in lung branching compared to the control cohort (OO) (Fig 2A). At E16.5 a clear diaphragmatic defect with intrathoracic migration of abdominal organs could be seen in more than 50% of the investigated N/B-exposed pups (Fig 2B).

### Nitrofen/bisdiamine results in lower lung weight/body weight ratios (LBWR) and histological lung hypoplasia

**Fig 3:**
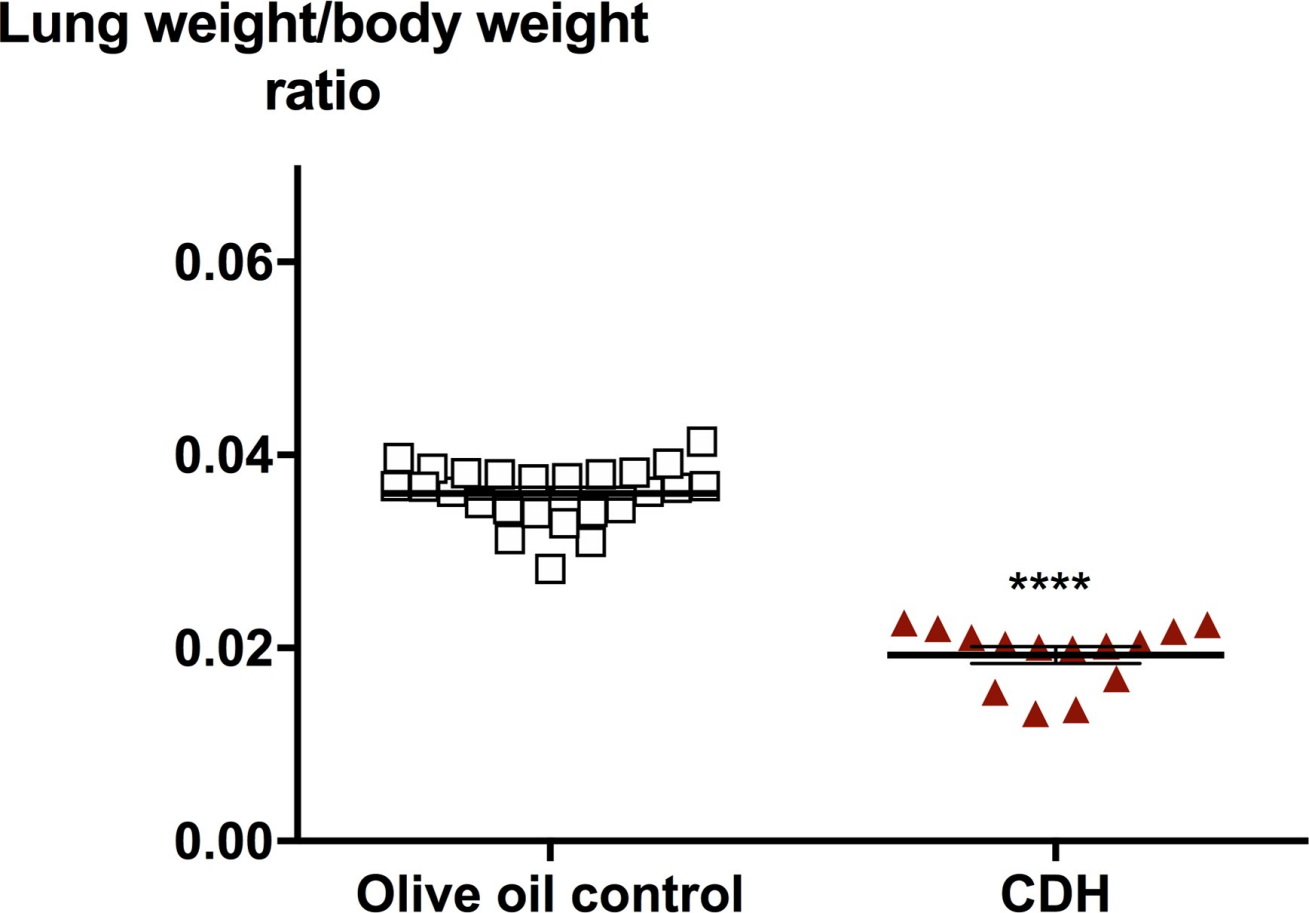
Congenital diaphragmatic hernia pups had lower lung weight/body weight ratio compared to the olive oil controls. Lung weight–to–body weight ratio was significantly decreased in the N/B-induced CDH group. Data indicate means ± SEM. N = 14–25. ****p < 0.0001, Student’s t-test.

**Fig 4:**
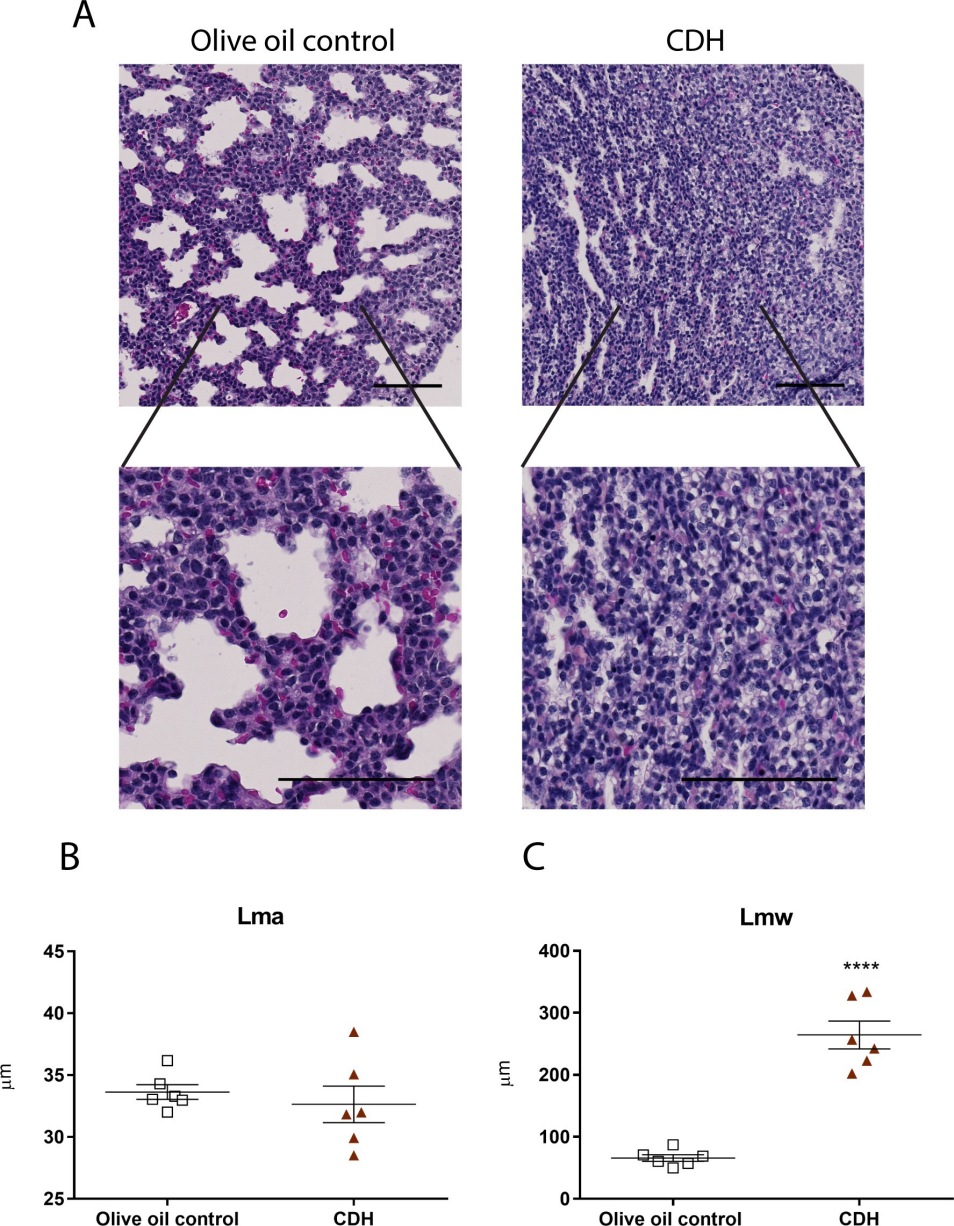
Congenital diaphragmatic hernia lungs showed thickened alveolar walls and increased interstitial tissue. (A) Representative histological images of control and CDH lungs revealed that CDH rodents exhibited wall thickening and rudimentary alveoli. Scale bars represent 100 μm. Morphometry analysis of mean linear intercept of parenchymal airspace (Lma) (B) and mean transactional wall length (Lmw) (C) showed an increase in the thickness of the septa of the parenchymal air-spaces. Data indicate means ± SEM. N = 6. ****p < 0.0001, Student’s t-test.

N/B-exposed mice had lung hypoplasia evidenced by lower LBWR in CDH lungs at E17.5, compared to controls (0.0193 ± 0.0008, n = 14 vs 0.0360 ± 0.0006, n = 25, p<0.0001) (Fig 3). Histologic assessment of E17.5 lungs revealed that CDH fetuses exhibit lungs with morphologic immaturity, including diminished alveolar airspaces, thickened alveolar walls and increased interstitial tissue (Fig 4A). Lung morphometry confirmed these observations as mean transactional wall length (Lmw) (p<0.0001) were significantly increased in N/B-CDH pups compared to controls (Fig 4B and 4C). We did not observe much difference in the fibrotic degree between CDH and controls using Masson's Trichrome staining (S6 Fig).

### AT1 cell markers are decreased and AT2 cell markers remain stable in CDH lungs

**Fig 5:**
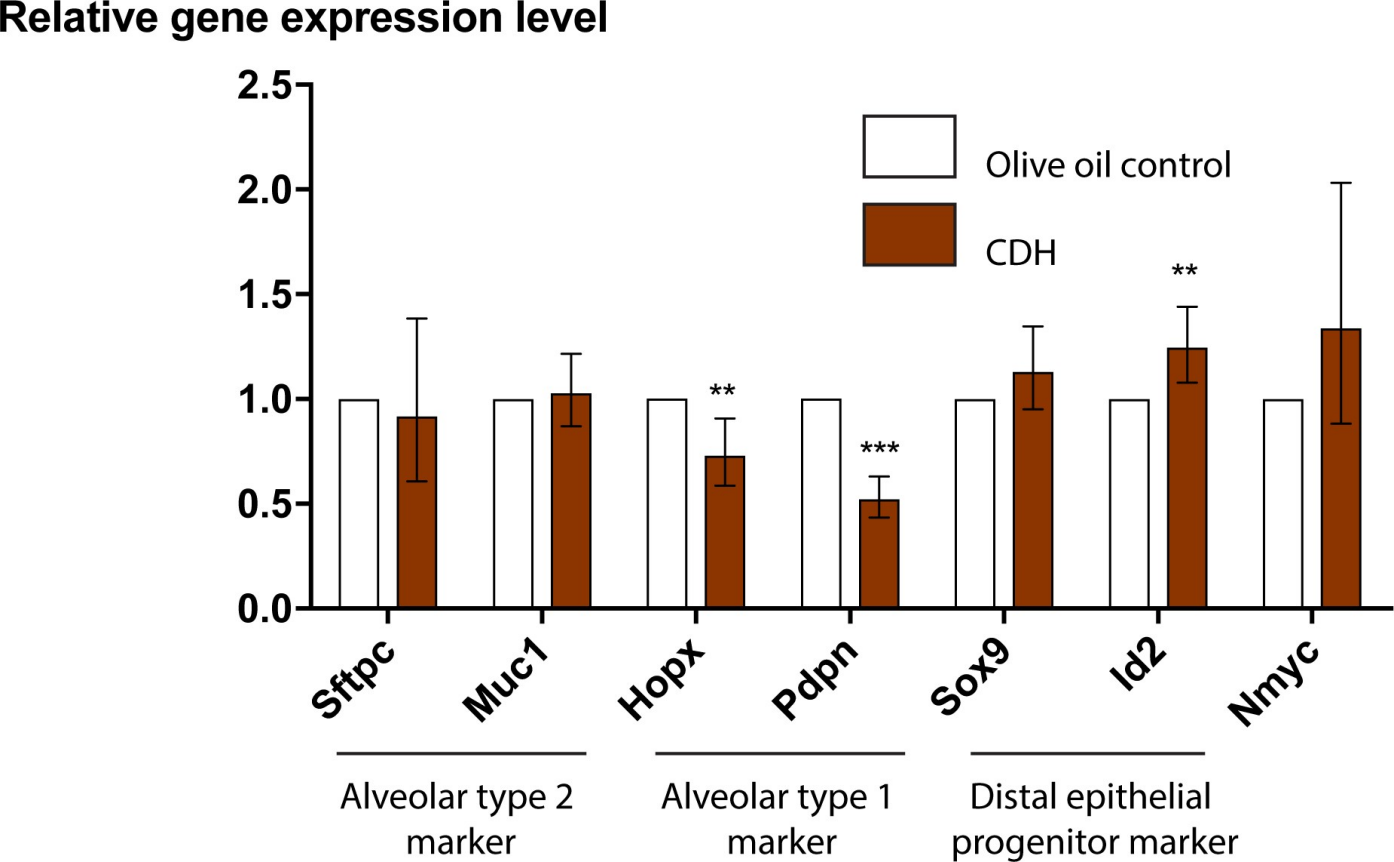
RT-qPCR analysis of congenital diaphragmatic hernia lungs showed an increase in distal epithelial progenitor and a decrease in type 1 alveolar cells. E17.5 CDH lungs demonstrated a decrease in mRNAs identifying alveolar epithelial type I cells (*Hopx*, *Pdpn*) while distal epithelial progenitor marker *Id2* was increased. Graph showing the mean values of fold change of E17.5 CDH relative to those of olive oil control lungs. Both CDH and control values were relative to those of the internal control gene *Hprt*, with CDH values representing the fold change relative to that of controls, which was converted to 1. For each gene, 6 pups were analyzed per group. Error bars represent the range of fold changes derived from ΔΔCt standard deviations (Fold change = 2^(-ΔΔCt)^). **p < 0.01, *** p < 0.001, Student’s t-test.

Real-time quantitative PCR (RT-qPCR) was performed on E17.5 lungs to assess the alveolar epithelial marker expression. Analysis of gene expression in mouse CDH lungs demonstrated that mRNAs associated with cell cycle progression (*Nmyc*) (p = 0.126) and those associated with differentiated AT2 cells (*Sftpc*, *Muc1*) (p = 0.624 and p = 0.698) were not different from the controls. However, distal epithelial progenitor markers *Id2* (fold change 1.25, range 1.08–1.44) was increased (p<0.01). More significantly, mRNAs typically associated with AT1 cells (*Hopx*, *Pdpn*) were significantly decreased in CDH pups (*Hopx* fold-change 0.73, range 0.58–0.90) (*Pdpn* fold-change 0.52, range 0.43–0.63) (p<0.01 and p<0.0001) (Fig 5).

### AT1 (Pdpn^+^) cell numbers are reduced while AT2 (proSPC^+^) cell numbers remain constant in CDH lungs

**Fig 6:**
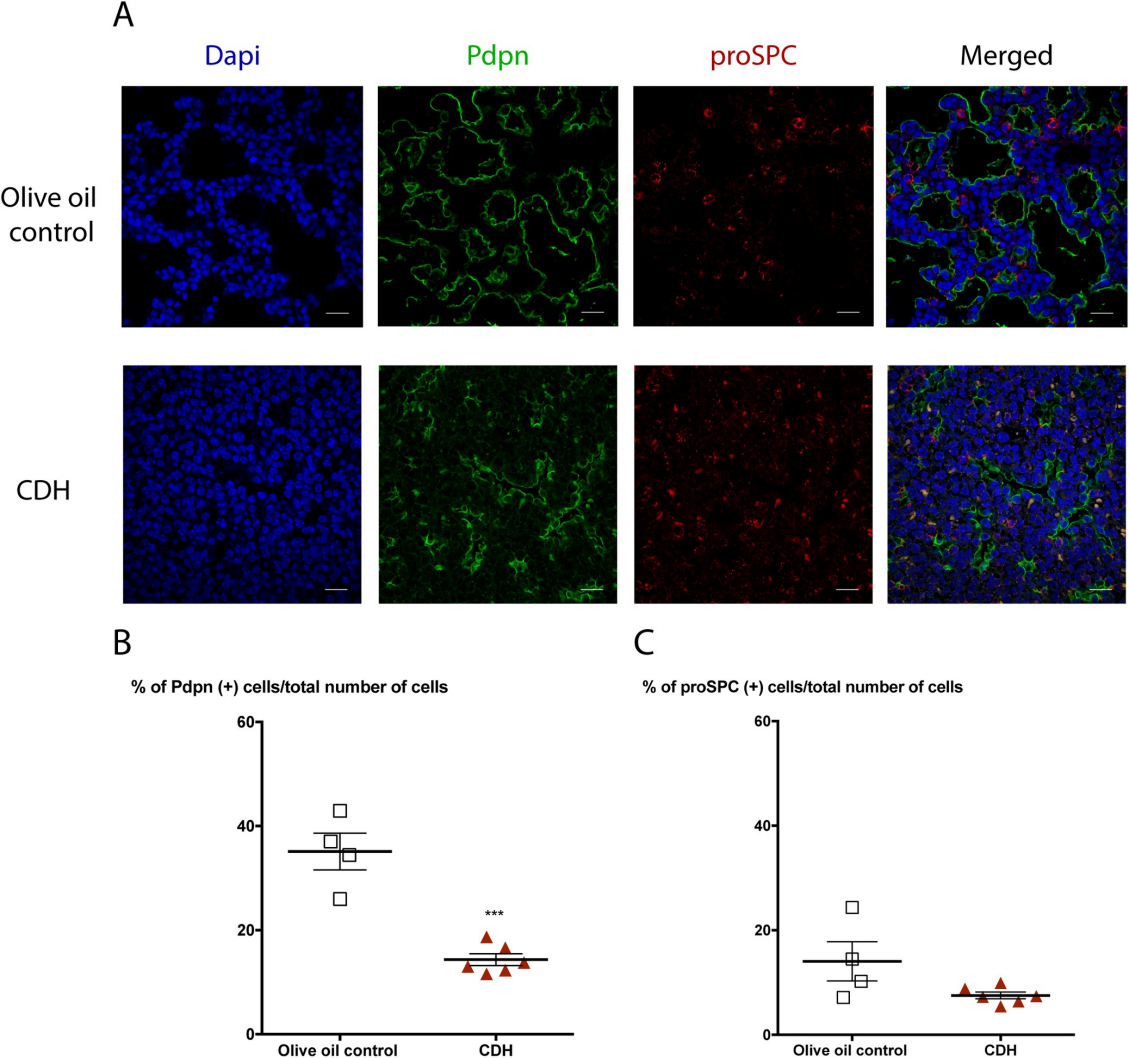
Nitrofen/bisdiamine-induced congenital diaphragmatic hernia lungs showed a decrease in the number of AT1 cells and no change in AT2 cells. Confocal representative images (A) and quantification of olive oil control (n = 4) and CDH lungs (n = 6) with antibodies to Pdpn (green) (B) and pro-SPC (red) (C) and showed a significant decline in the population of AT1 Pdpn^+^ cells. Nuclei stained with DAPI (blue). Scale bars represent 20 μm. Total number of cells counted per sample is higher than 1000 cells. Data shown are means ± SEM. ***p < 0.001, Student’s t-test.

To determine whether the change in gene expression of AT1 and AT2 markers reflects a modification at the protein level in N/B-induced CDH lungs, immunofluorescence was performed in E17.5 lungs. Staining for AT2 marker proSPC was combined with the AT1 marker Pdpn in order to evaluate their expression and localization at E17.5 (S3 Fig). Cells with both markers were counted as BPs. Confocal microscopy corroborated the RT-qPCR results by showing a marked decrease in Pdpn expression in the distal alveolar epithelium of CDH lungs while proSPC expression was the same in both groups (Fig 6A). Quantitively, the percentage of Pdpn^+^ cells was lower in the lungs from N/B CDH animals (35.1 ± 3.522, n = 4 in controls; 14.33 ± 1.122, n = 6 in CDH) (p<0.001), whereas the number of proSPC^+^ cells was not significantly different (14.05 ± 3.75, n = 4 in controls; 7.539 ± 0.6618, n = 6 in CDH) (p = 0.0672) (Fig 6B and 6C). The decrease of AT1 cells in CDH lungs was confirmed by immunofluorescence staining for another AT1 marker Hopx^+^ (S5 Fig). The percentage of the BP (Pdpn^+^/proSPC^+^) was not significantly different between the CDH and control groups (3.22 ± 0.5905, n = 4 in controls; 2.182 ± 0.2574, n = 6 in CDH, p = 0.1044) (S4 Fig).

## Discussion

**Fig 7:**
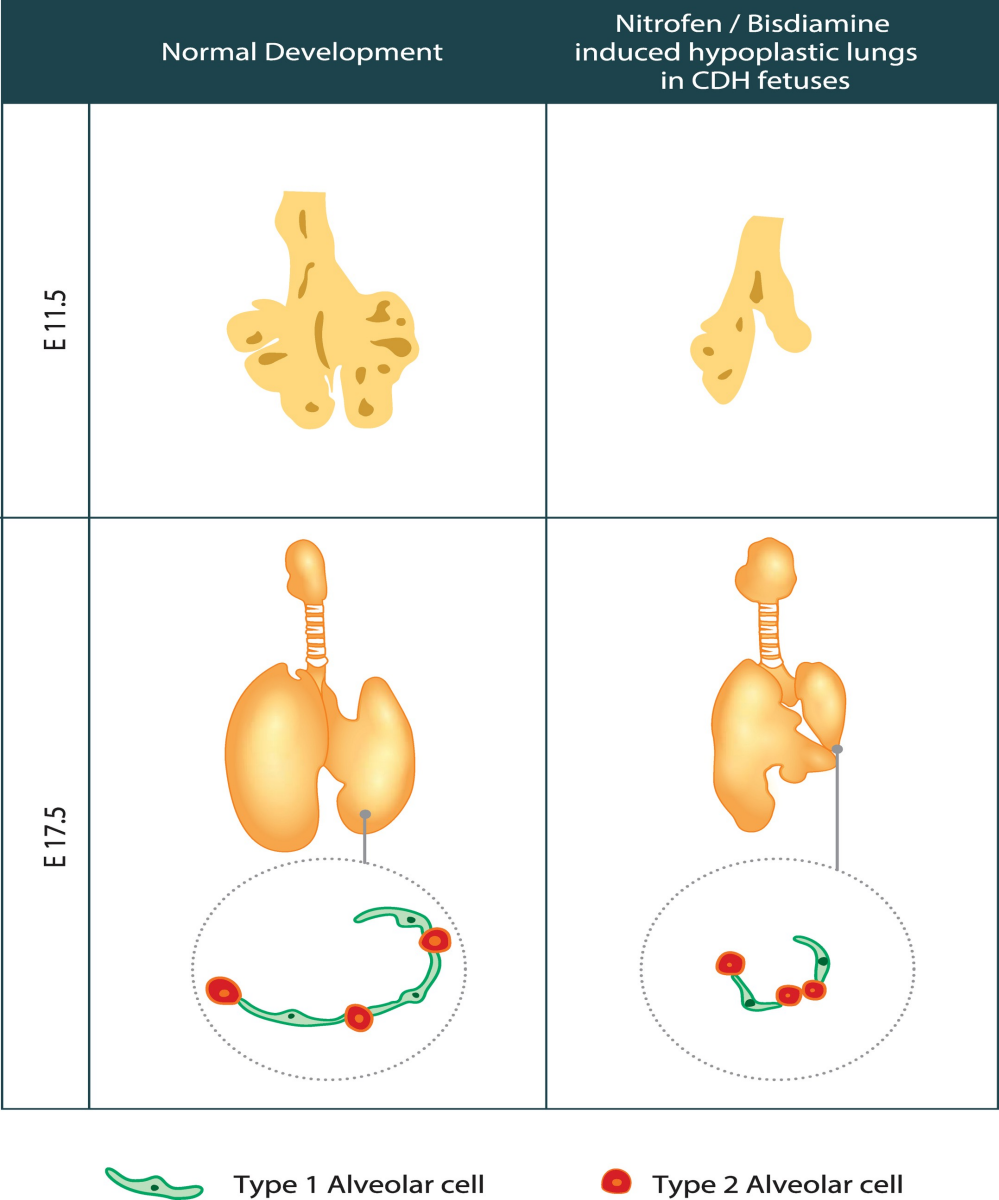
Graphical summary of our findings. Nitrofen/bisdiamine-induced congenital diaphragmatic hernia fetuses showed delayed lung development from the embryonic stage (E11.5). At late canalicular stage (E17.5), the expression of AT1 markers in the CDH lungs were decreased compared to the controls while AT2 markers were not significantly altered.

Herein, we used a mouse teratogenic model of CDH by N/B induction to further elucidate the status of AEC differentiation in hypoplastic lungs associated with CDH. N/B-induced CDH fetuses showed lung hypoplasia with lower LBWR and thickened alveolar wall, in association with early lung delay shown by nanoCT analysis. The expression of AT1 markers in the CDH lungs were significantly decreased while AT2 markers remained unchanged at both gene expression and protein levels compared to the controls (Fig 7).

We were interested to see if the mouse teratogenic model displays a clinical CDH phenotype similar to the rat nitrofen model. N/B administration in mice results in lung hypoplasia as early the embryonic phase and later a diaphragmatic defect with migration of abdominal organs into the thoracic cavity at canalicular stage resembling the rat model and human condition [24,34–36]. Lung hypoplasia was also observed using validated outcome measures including LBWR and pulmonary morphometry. LBWR was significantly lower in the N/B-induced CDH fetuses compared to controls, which parallels CDH rabbit and rat models [37–42]. At E17.5 in mouse pups, corresponding to human week 24–26, the LBWR of the CDH lungs was at 0.0193, which also agrees with postmortem studies where an indication of pulmonary underdevelopment is based on a LBWR from lower than 0.015 to 0.022 [43,44].

The CDH mouse model allowed us to gain a better insight into CDH lung development as we could use a much broader range of assessments including whole-fetus nanoCT, RT-qPCR and immunofluorescence co-staining of multiple markers of interest. In addition to the left sided herniation and lung hypoplasia at E16.5, nanoCT revealed that lung development was delayed as early as the embryonic stage E11.5 when the diaphragm has not finished its formation. This is in line with other teratogenic CDH models where abnormal lung development occurs independently of those resulting secondarily to the diaphragm defects [45–48]. Lung histology confirms the delay in lung development in CDH lungs with morphologic immaturity in combination with increased medial wall thickness similar to other models [37,38,40,49].

The status of AECs differentiation in hypoplastic lungs associated with CDH is still unclear and controversial, both in human and in animal models [23,50–52]. We determined the relative levels of AT1 and AT2 by RT-qPCR and immunofluorescence imaging. Our observation is aligned with previous findings showing that AT1 cell density is decreased in CDH lungs [23,24,53]. One possible explanation would be that AT1 cells trans-differentiate into AT2 in CDH lungs [54,55]. However, if this is the case, one would expect an increase in the AT2 population. Yet, our results suggest no significant difference in the number of AT2 number in CDH lungs. Another possibility is that the differentiation from the BP cell towards AT1 is inhibited due to a decrease in CDH lung parenchyma stretch as mechanical forces have been shown to be required for AT1 cell differentiation [56].

As the number of AT2 cells remained unchanged in our CDH pups, our results disagreed with previous studies where the number of AT2 cells and their surfactant production were found to be deficient in CDH [23,24,51,52]. There may be several reasons for these disparate results. One possibility is that previous studies analyzed AEC differentiation status before BP cells were extensively described. This could induce counting errors as these progenitors express both AT1 and AT2 markers. As most of these studies evaluated the number of AT2 and AT1 cells based on light microscopic morphology or separate staining of proSPC and Pdpn, identification errors of cell types cannot be excluded. In our study, we performed co-staining for markers that allowed us to discriminate AT1, AT2 and BP, and count them separately. Thus, our results might represent a more accurate evaluation of AT1 and AT2 ratios in CDH lungs. Our results are also in line with studies where no benefit associated with surfactant therapy was found for term infants with a prenatal diagnosis of isolated CDH [57,58].

A limitation of our model is the potential developmental effect of N/B teratogenicity on the fetal mice. Unlike the rat model, the teratogenic effects in mice seem to be broader and maxillofacial abnormalities were noticed. It remains unclear if these abnormalities contribute to the effects seen on lung development. However, as abnormalities do not occur in all individuals and a wide array of developmental assessments can be used, this model still provides valuable information with regard to alveolar epithelial differentiation status in CDH-induced lung hypoplasia.

In conclusion, we have shown that the mouse teratogenic model is an elegant model that accommodates visualizing CDH lung development in great depth at early gestational ages. It has potential for wider use in the study of the pathogenesis of CDH, given the cornucopia of molecular methods such as transcriptomics and proteomics. We detected a decrease in AT1 cells in the late canalicular stage of lung development in CDH lungs while AT2 cells number was not significantly different. To identify which of the aforementioned scenarios is the true origin of this change, further research where lineage tracing can be used to follow the BPs choice of differentiation into either AT1 or AT2 in CDH lungs is required. This will lead to a greater understanding of CDH etiology, therefore aiding in identifying novel therapeutic targets in the near future.

## Supporting information

S1 Table Primers used in RT-qPCR. (PDF)

S2 Table Abnormalities observed in E16.5 nitrofen/bisdiamine exposed pups using nanoCT. (PDF)

S1 Fig Diaphragmatic defect in E17.5 pups. Nitrofen/bisdiamine administration at E8.5 creates a proportion of pups with diaphragmatic defects. Defect on the diaphragm is marked by the dashed circle.
(PNG)

S2 Fig Different doses of bisdiamine affected the survival, congenital diaphragmatic hernia and facial abnormality rates per mother. (A) The survival rate was calculated by number of survived pups/ number of sacs detected per mother on harvesting day. (B) CDH rate was calculated by number of CDH pups (with visible diaphragmatic defect and herniation)/number of survived pups. (C) Facial abnormality rate was calculated by number of pups with facial abnormality/number of survived pups. Each dot represents a single mom. Bars represent means ± SEM. (D) Representative image of abnormal face in an E16.5 pup vs an olive oil control pup.
(TIF)

S3 Fig Identification of alveolar type 1 and alveolar type 2 cells. (PNG)

S4 Fig Percentage of Pdpn^+^proSPC^+^ bipotent progenitors in CDH vs normal lungs. (PNG)

S5 Fig Percentage of Hopx+ AT1 cells in CDH vs normal lungs. Confocal representative images (A) and quantification of olive oil (n = 4) and CDH lungs (n = 6) with antibodies to Hopx (red), Pdpn (green) and pro-SPC (blue) confirmed the decline in the population of AT1 cells in CDH lungs. Nuclei stained with DAPI (white). Scale bar represent 20um. Total number of cells counted per sample is higer than 1000 cells. Data shown are means ± SEM. **p < 0.01, Student’s t-test.
(TIF)

S6 Fig Histochemical staining for fibrosis by Masson's trichrome stain in CDH and normal control lungs. (TIF)

S1 File A 3d-reconstructed fetus with congenital diaphragmatic hernia from nanoCT scan. (MP4)

S2 File A 3d-reconstructed olive oil control fetus from nanoCT scan. (MP4)
