# Correction: The proportion of alveolar type 1 cells decreases in murine hypoplastic congenital diaphragmatic hernia lungs

**DOI:** 10.1371/journal.pone.0217322

**Published:** 2019-07-03

**Authors:** Tram Mai Nguyen, Julio Jimenez, Linda Elowsson Rendin, Catharina Müller, Gunilla Westergren-Thorsson, Jan Deprest, Jaan Toelen

S4 Fig appears incorrectly. The correct S4 Fig can be viewed below.

## Supporting information

S4 Fig Percentage of Pdpn^+^proSPC^+^ bipotent progenitors in CDH vs normal lungs. (TIFF)
