# Supplementary material for: The proportion of alveolar type 1 cells decreases in murine hypoplastic congenital diaphragmatic hernia lungs
Source: PLoS One. 2019 Apr 17;14(4):e0214793. doi: 10.1371/journal.pone.0214793 (PMC6469843; doi:10.1371/journal.pone.0214793)
Supplement: S1 Table — (PDF) [file pone.0214793.s001.pdf]

Gene name	Forward primer 5’– 3’	Reverse primer 3’– 5’
Pdpn	ACCGTGCCAGTGTTGTTCTG	AGCACCTGTGGTTGTTATTTTGT
Hopx	TTCAACAAGGTCAACAAGCACCCG	CCAGGCGCTGCTTAAACCATTTCT
Muc1	GGCTCCGTGGTGGTAGAATC	CAGAGGGAGGGAACTGCATC
Id2	GACCCGATGAGCCTGCTATAC	AATAGTGGGATGCGAGTCCAG
Sox9	AGGAAGCTGGCAGACCAGTA	TCCACGAAGGGTCTCTTCTC
Sftpc	ATGGACATGAGTAGCAAAGAGGT	CACGATGAGAAGGCGTTTGAG
Nmyc	AGCACCTCCGGAGAGGATA	TCTCTACGGTGACCACATCG
Hprt	CTCATGGACTGATTATGGACAGGAC	GCAGGTCAGCAAAGAACTTATAGCC
