# Supplementary material for: The proportion of alveolar type 1 cells decreases in murine hypoplastic congenital diaphragmatic hernia lungs
Source: PLoS One. 2019 Apr 17;14(4):e0214793. doi: 10.1371/journal.pone.0214793 (PMC6469843; doi:10.1371/journal.pone.0214793)
Supplement: S2 Table — (PDF) [file pone.0214793.s002.pdf]

Pup	Treatment	Heart abnormality	Brain abnormality	Other abnormalities
1	Nitrofen	Yes	Yes	Diaphragm; Lung; Facial; Renal
2	Nitrofen	Yes	N.A.	Diaphragm; Lung; Facial;
3	Nitrofen	Yes	No	Diaphragm; Lung
4	Nitrofen	Enlarged	No	Diaphragm; Lung
5	Nitrofen	Enlarged	No	Lung
6	Nitrofen	Yes	No	Diaphragm; Lung
7	Nitrofen	Yes	No	Diaphragm; Lung
8	Nitrofen	Enlarged	No	Lung; Renal
9	Nitrofen	Yes	No	Diaphragm; Lung; Renal
10	Nitrofen	Yes	Yes	Diaphragm; Lung; Facial
11	Nitrofen	Enlarged	No	Lung
12	Olive oil	No	No	None
13	Olive oil	No	No	None
14	Olive oil	No	No	None
