# Supplementary figures and images for: The proportion of alveolar type 1 cells decreases in murine hypoplastic congenital diaphragmatic hernia lungs
Source: PLoS One. 2019 Apr 17;14(4):e0214793. doi: 10.1371/journal.pone.0214793 (PMC6469843; doi:10.1371/journal.pone.0214793)

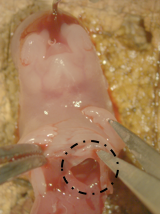

Supplement: S1 Fig — Nitrofen/bisdiamine administration at E8.5 creates a proportion of pups with diaphragmatic defects. Defect on the diaphragm is marked by the dashed circle. (PNG) [file pone.0214793.s003.png]

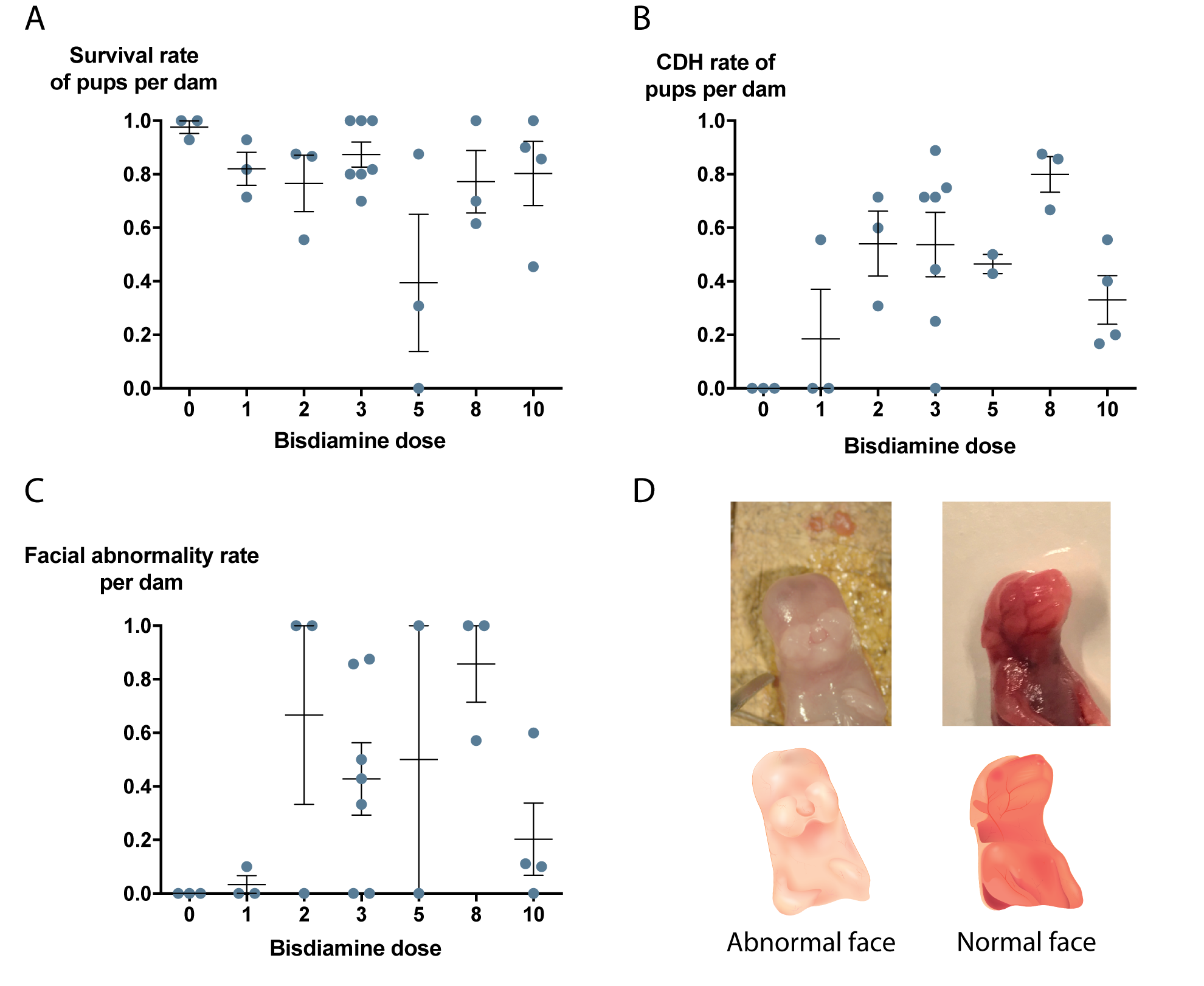

Supplement: S2 Fig — (A) The survival rate was calculated by number of survived pups/ number of sacs detected per mother on harvesting day. (B) CDH rate was calculated by number of CDH pups (with visible diaphragmatic defect and herniation)/number of survived pups. (C) Facial abnormality rate was calculated by number of pups with facial abnormality/number of survived pups. Each dot represents a single mom. Bars represent means ± SEM. (D) Representative image of abnormal face in an E16.5 pup vs an olive oil control pup. (TIF) [file pone.0214793.s004.tif]

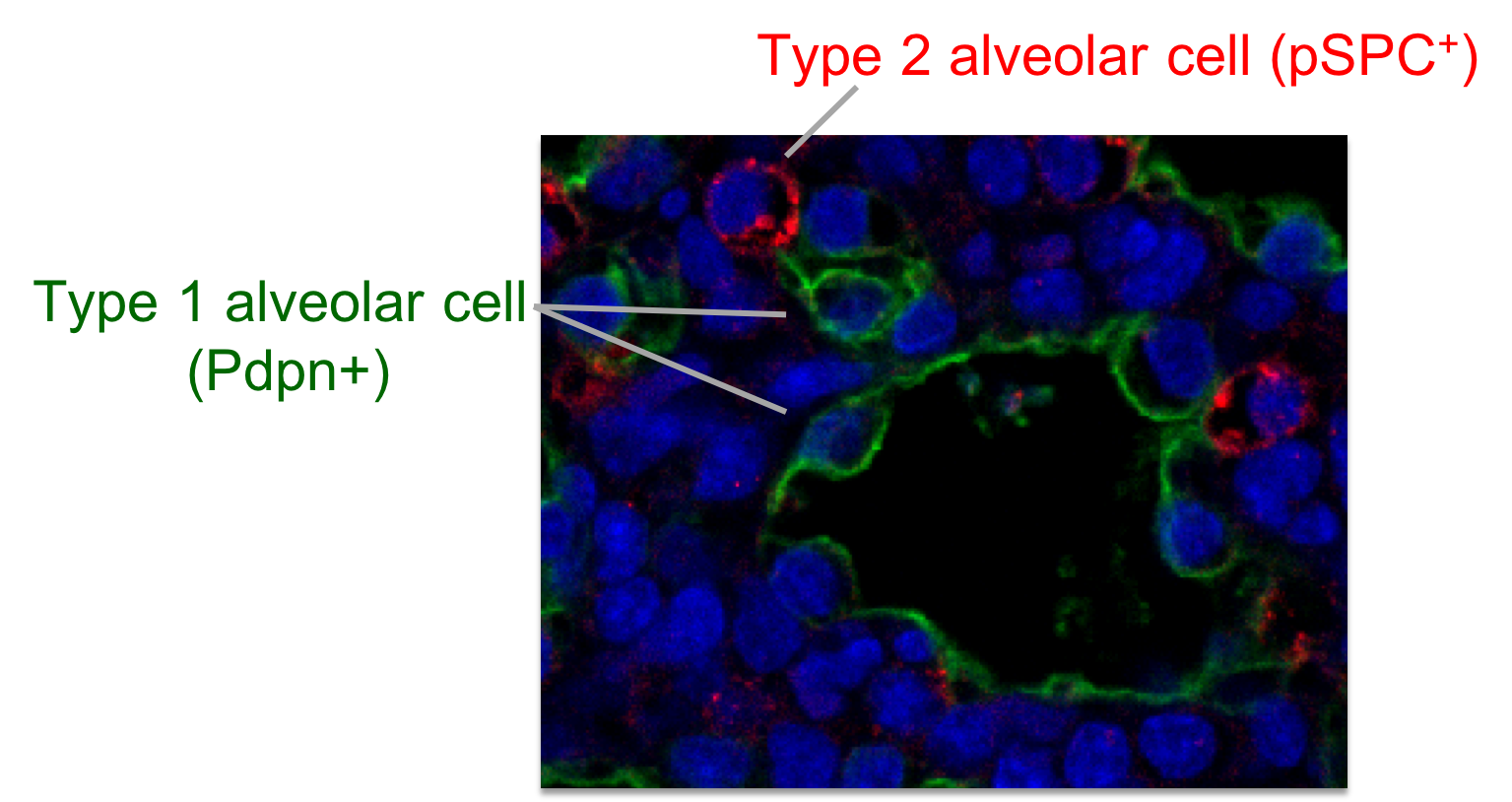

Supplement: S3 Fig — (PNG) [file pone.0214793.s005.png]

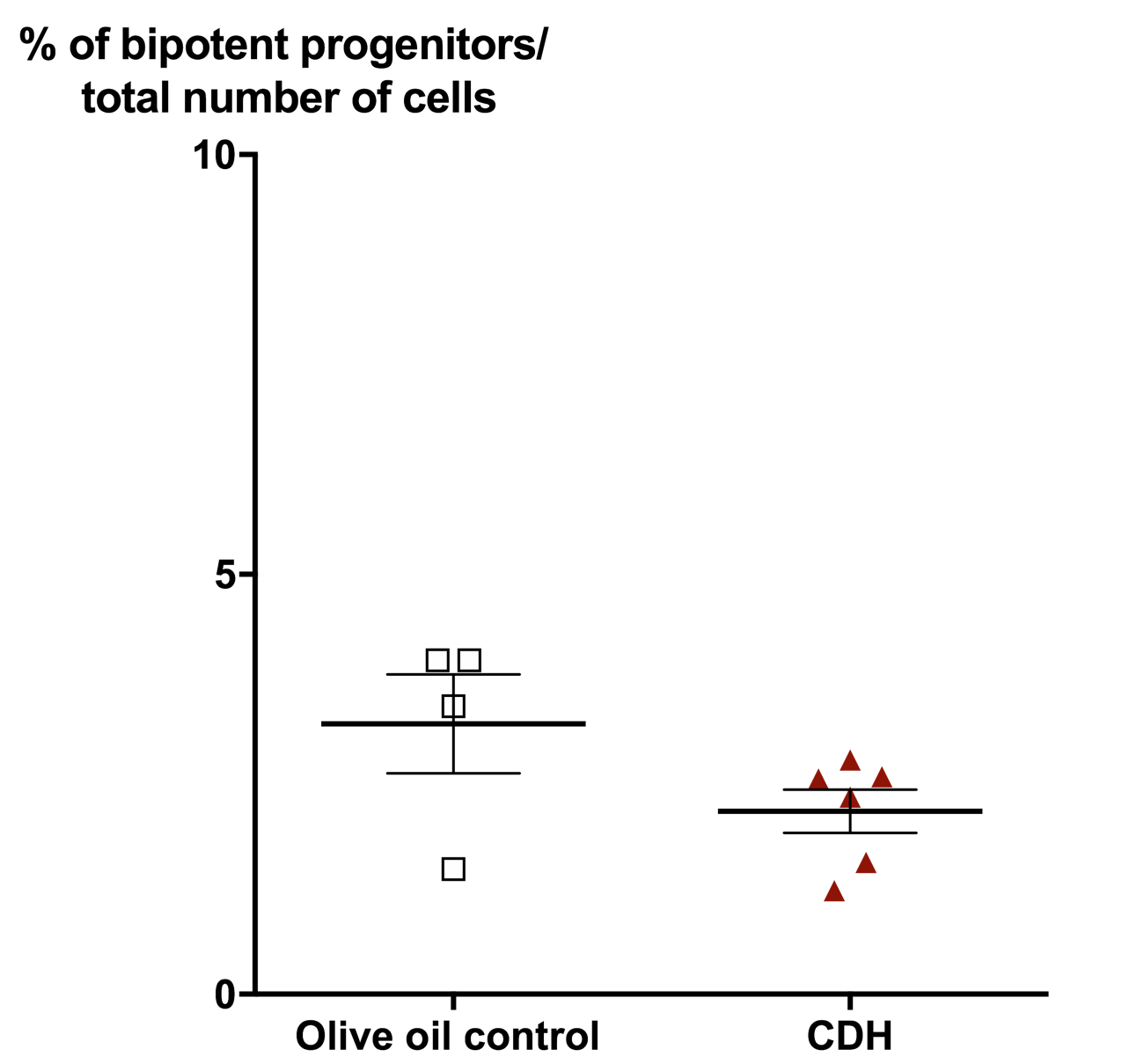

Supplement: S4 Fig — (PNG) [file pone.0214793.s006.png]

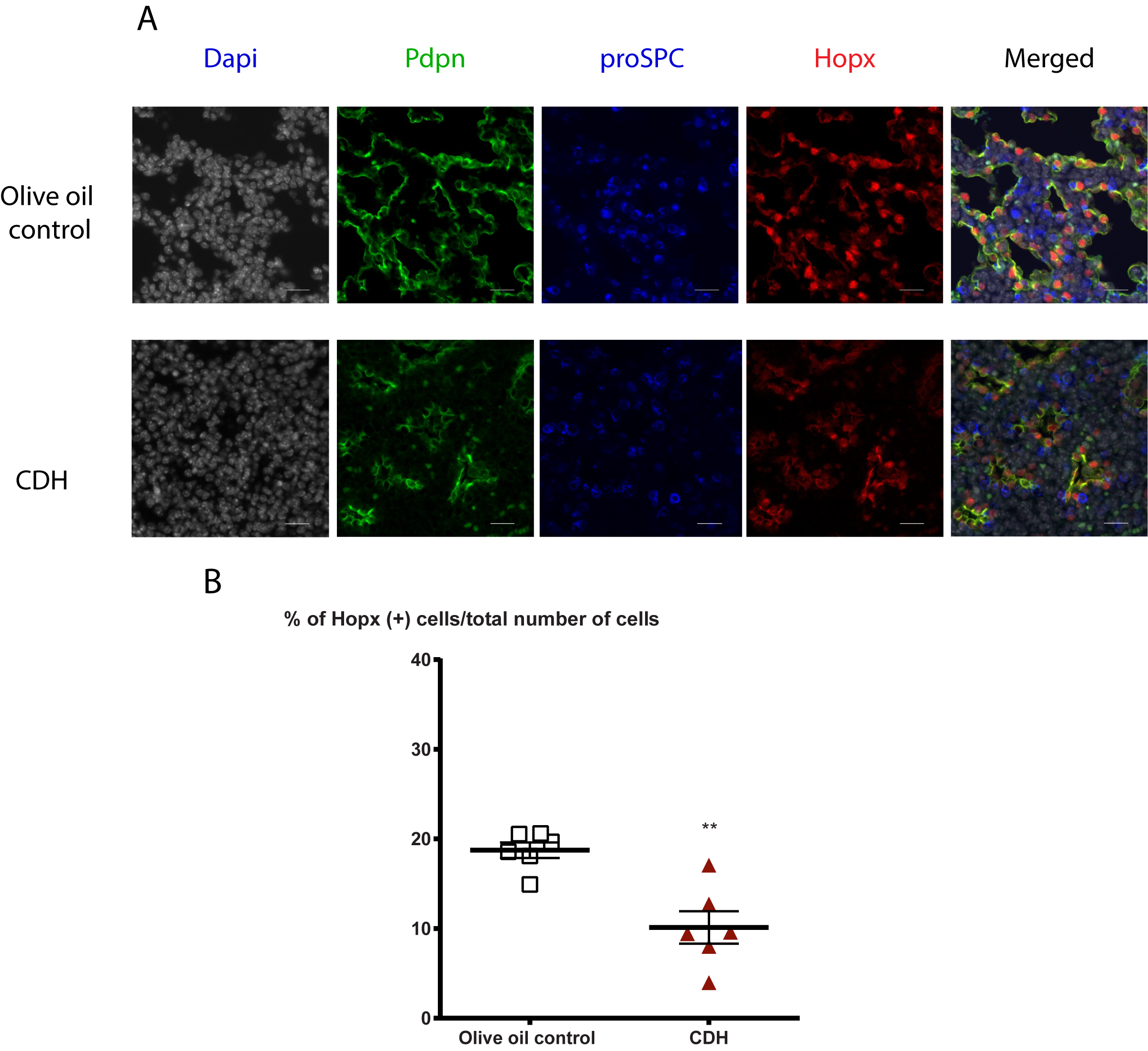

Supplement: S5 Fig — Confocal representative images (A) and quantification of olive oil (n = 4) and CDH lungs (n = 6) with antibodies to Hopx (red), Pdpn (green) and pro-SPC (blue) confirmed the decline in the population of AT1 cells in CDH lungs. Nuclei stained with DAPI (white). Scale bar represent 20um. Total number of cells counted per sample is higer than 1000 cells. Data shown are means ± SEM. **p < 0.01, Student’s t-test. (TIF) [file pone.0214793.s007.tif]

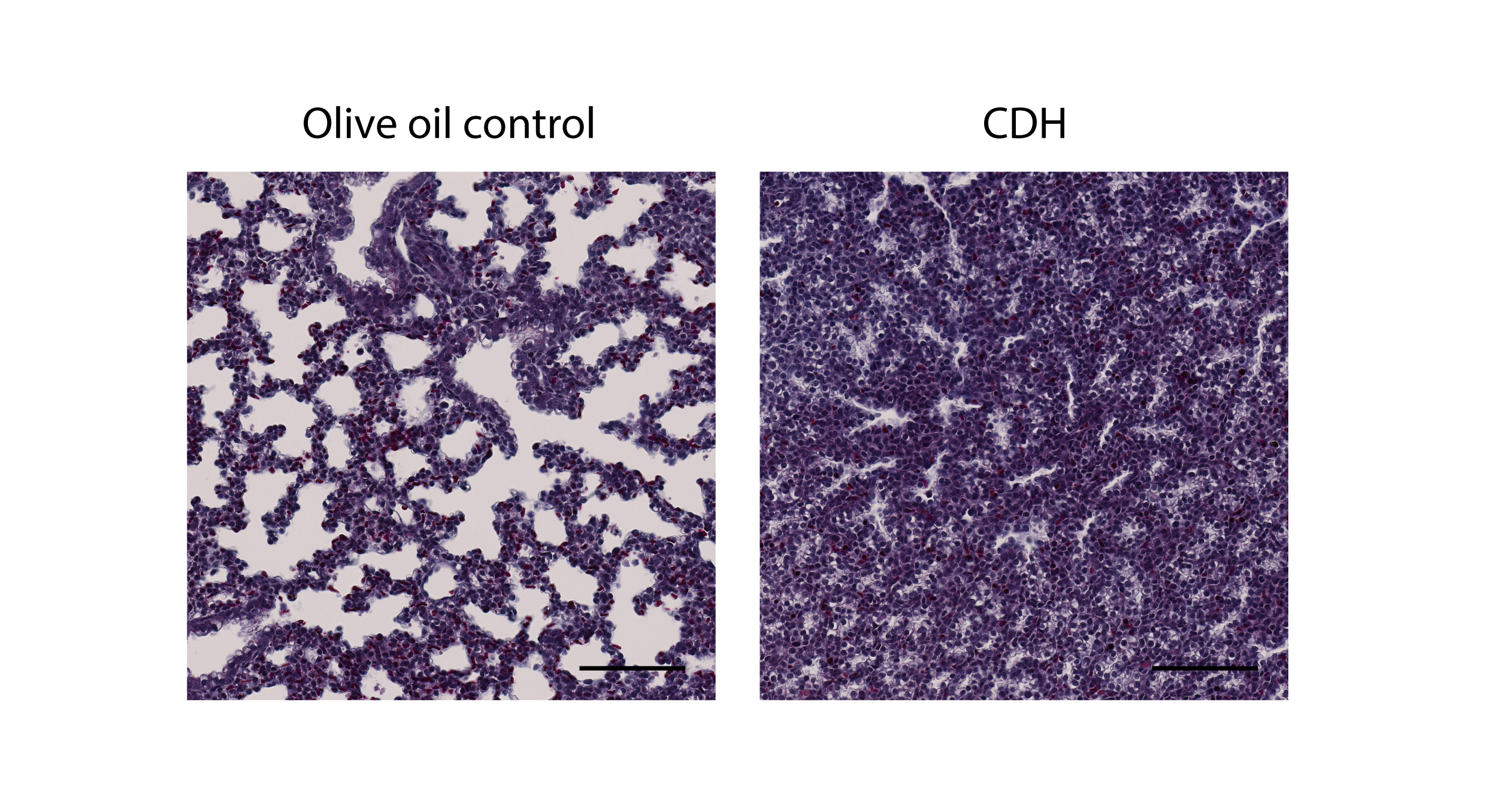

Supplement: S6 Fig — (TIF) [file pone.0214793.s008.tif]
