# Supplementary figures and images for: Correction: The proportion of alveolar type 1 cells decreases in murine hypoplastic congenital diaphragmatic hernia lungs
Source: PLoS One. 2019 Jul 3;14(7):e0217322. doi: 10.1371/journal.pone.0217322 (PMC6608939; doi:10.1371/journal.pone.0217322)

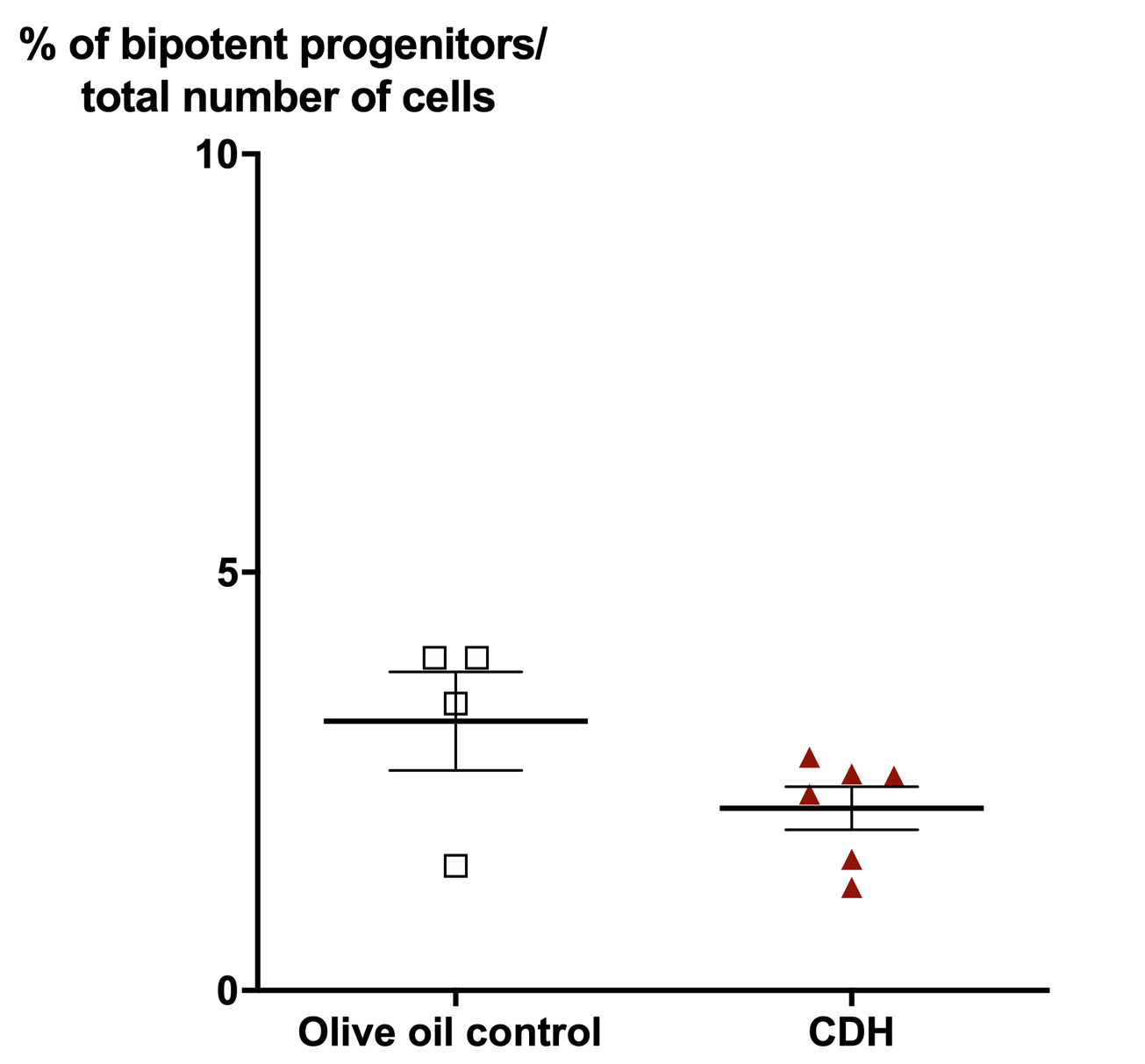

Supplement: S4 Fig — (TIFF) [file pone.0217322.s001.tiff]
